# Networking of glucagon-like peptide-1 axons with GnRH neurons in the basal forebrain of male mice revealed by 3DISCO-based immunocytochemistry and optogenetics

**DOI:** 10.1007/s00429-020-02167-7

**Published:** 2020-11-09

**Authors:** Csaba Vastagh, Imre Farkas, Michael M. Scott, Zsolt Liposits

**Affiliations:** 1 Laboratory of Endocrine Neurobiology, Institute of Experimental Medicine, Szigony u. 43, 1083 Budapest, Hungary; 2 Laboratory of Reproductive Neurobiology, Institute of Experimental Medicine, Budapest, Hungary; 3 Department of Pharmacology, School of Medicine, University of Virginia, Charlottesville, VA USA; 4 Department of Neuroscience, Faculty of Information Technology and Bionics, Pázmány Péter Catholic University, Budapest, Hungary

**Keywords:** Glucagon-like peptide-1, GnRH neuron, Neuronal networking, 3DISCO, Optogenetics, Slice electrophysiology, Patch clamp, Transgenic mice

## Abstract

Glucagon-like peptide-1 (GLP-1) regulates reproduction centrally, although, the neuroanatomical basis of the process is unknown. Therefore, the putative networking of the central GLP-1 and gonadotropin-releasing hormone (GnRH) systems was addressed in male mice using whole mount immunocytochemistry and optogenetics. Enhanced antibody penetration and optical clearing procedures applied to 500–1000 µm thick basal forebrain slices allowed the simultaneous visualization of the two distinct systems in the basal forebrain. Beaded GLP-1-IR axons innervated about a quarter of GnRH neurons (23.2 ± 1.4%) forming either single or multiple contacts. GnRH dendrites received a more intense GLP-1 innervation (64.6 ± 0.03%) than perikarya (35.4 ± 0.03%). The physiological significance of the innervation was examined by optogenetic activation of channelrhodopsin-2 (ChR2)-expressing axons of preproglucagon (GCG) neurons upon the firing of GnRH neurons by patch clamp electrophysiology in acute brain slices of triple transgenic mice (Gcg-cre/ChR2/GFP-GnRH). High-frequency laser beam stimulation (20 Hz, 10 ms pulse width, 3 mW laser power) of ChR2-expressing GCG axons in the mPOA increased the firing rate of GnRH neurons (by 75 ± 17.3%, *p* = 0.0007). Application of the GLP-1 receptor antagonist, Exendin-3-(9-39) (1 μM), prior to the photo-stimulation, abolished the facilitatory effect. In contrast, low-frequency trains of laser pulses (0.2 Hz, 60 pulses) had no effect on the spontaneous postsynaptic currents of GnRH neurons. The findings indicate a direct wiring of GLP-1 neurons with GnRH cells which route is excitatory for the GnRH system. The pathway may relay metabolic signals to GnRH neurons and synchronize metabolism with reproduction.

**Electronic supplementary material:**

The online version of this article (10.1007/s00429-020-02167-7) contains supplementary material, which is available to authorized users.

## Introduction

Glucagon-like peptide-1 (GLP-1) (Kreymann et al. 1987), a cleavage product of the preproglucagon peptide encoded by the *Gcg* gene, is a potent hormone produced by intestinal L-cells (Baggio and Drucker 2007; Mojsov et al. 1990) and neurons residing in the nucleus of the solitary tract (NTS) and the reticular nucleus of the medulla oblongata (Larsen et al. 1997; Merchenthaler et al. 1999; Vrang and Larsen 2010). The hormone is capable of reducing food intake (Hayes et al. 2008; Kinzig et al. 2002), inhibiting gastric emptying (Chelikani et al. 2005), and increasing glucose-stimulated insulin secretion (Knauf et al. 2005; Komatsu et al. 1989; Drucker 1998). The GLP-1 producing neurons of the lower brain stem give rise to rich axon projections innervating functionally important loci of the neuroaxis like the hypothalamus, thalamus, septal regions, cerebral cortex and different nuclei of hindbrain (Trapp and Cork 2015). Accordingly, its G-protein-coupled receptor (GLP-1R) is also widely expressed in the brain including centers regulating homeostasis (Goke et al. 1995; Scrocchi et al. 1996; Wei and Mojsov 1995; Cork et al. 2015; Li et al. 2003; Richard et al. 2014; Richards et al. 2014; Sandoval and Sisley 2015; Sandoval et al. 2008). In a recent report, the chemogenetic activation of preproglucagon (GCG) synthesizing neurons of the NTS has been shown to reduce food intake and body weight via their communication with downstream homeostatic regulatory centers (Gaykema et al. 2017).

GLP-1 has also been identified as a modulator of the hypothalamo-pituitary-gonadal (HPG) axis. A previous study (Beak et al. 1998) has explored that GLP-1 administration evokes a concentration-dependent increase in gonadotropin-releasing hormone (GnRH) release from the immortalized GnRH-producing GT1-7 neurons (Wetsel et al. 1992; Mellon et al. 1990) and its intracerebroventricular injection increases the plasma luteinizing hormone (LH) level of male rats (Beak et al. 1998). Furthermore, knocking out the GLP-1 receptor (GLP-1R) in mice resulted in reduced gonadal weights in males and a slight delay in the onset of puberty in females (MacLusky et al. 2000). Previous in vivo studies have elucidated the role of GLP-1 in increasing the amplitude of the preovulatory LH surge, regulating estradiol and progesterone levels and augmenting the number of Graafian follicles and corpora lutea (Outeirino-Iglesias et al. 2015). We have recently addressed the effects of GLP-1 receptor activation on the electrophysiological properties of GnRH neurons in acute brain slice preparations (Farkas et al. 2016). These studies elucidated that the potent GLP-1 receptor agonist, Exendin 4, elevated the firing rate of GnRH neurons and increased the frequency of miniature GABAergic postsynaptic currents (mPSCs) in GnRH cells. Regarding the downstream molecular cascades, the activation of GLP-1R facilitates the nitric oxide (NO) and suppresses the endocannabinoid retrograde signaling pathways, both mechanisms are known to target the excitatory, presynaptic GABA-ergic terminals (Farkas et al. 2010, 2016; Moenter and DeFazio 2005). GLP-1 receptors expressed in GnRH neurons (Farkas et al. 2016) may receive the hormonal signal from two distinct anatomical sources, the gut and the brain. The regulatory actions of brain-derived GLP-1 on GnRH neurons presume the networking of the central GCG neuronal system with GnRH neurons. However, the fine structural details, the intensity and the physiological significance of networking have not been elucidated yet.

The present study was undertaken to shed light on the structural characteristics of communication between the central GLP-1 and GnRH neuron systems and explore its physiological role. The simultaneous immunofluorescent detection of GLP-1 and GnRH immunoreactive (IR) elements in combination with the recently invented 3DISCO (3-Dimensional Imaging of Solvent-Cleared Organs) technique (Erturk et al. 2012) allowed the visualization, 3-dimensional (3D) reconstruction and quantitative analysis of the interacting neuronal systems in thick (500–1000 mm) brain slices. Optogenetics and patch clamp electrophysiology—performed on acute basal forebrain slices from a triple transgenic mouse line—were used to address the functional correlates of the explored networking. The results collectively indicate that a subset of GnRH neurons receives a substantial GLP-1-IR innervation arising from the medulla oblongata and optogenetic stimulation of these specific afferents evokes GLP-1 release that, in turn, modifies the firing pattern of GnRH neurons.

## Materials and methods

### Ethics statement

All animal studies were carried out with permissions from the Animal Welfare Committee of the Institute of Experimental Medicine (Permission Number: A5769-01) and in accordance with legal requirements of the European Community (Directive 2010/63/EU). All animal experimentation described was conducted in accord with accepted standards of humane animal care and all efforts were made to minimize suffering.

### Animals

Adult, male transgenic mice on the C57Bl/6 J or Bl6Fx background were used from local colonies bred at the Medical Gene Technology Unit of the Institute of Experimental Medicine (IEM). Three transgenic mouse lines were used as follows: *Mouse line 1*. GnRH-GFP mouse: green fluorescence protein (GFP) reporter was genetically targeted to GnRH neurons (Suter et al. 2000b). *Mouse line 2*. GCG-ChR2 mouse: *Gcg-Cre*) mice (Gaykema et al. 2017) were crossed with channelrhodopsin 2 (ChR2) reporter mice {Ai32[RCL-ChR2(H134R)/EYFP]} (Madisen et al. 2012). *Mouse line 3*. GCG-ChR2-GnRH-GFP mice: mouse line 2 - expressing ChR2 in GCG neurons of the NTS and reticular nucleus of the brain stem—were crossed with GnRH-GFP mice. For anatomical studies, the GnRH-GFP and GCG-ChR2 mouse lines were used. The GCG-ChR2-GnRH-GFP mouse line was invented for the purpose of optogenetic studies addressing the regulatory role of neuronal GLP-1 upon the electrophysiological activity of GnRH neurons.

All animals were housed in light (12:12 light–dark cycle, lights on at 06:00 h)—and temperature (22 ± 2 °C) controlled environment, with free access to standard food and tap water.

### Characterization of the transgenic mouse strains

#### The GnRH-GFP mouse line

The GnRH-GFP transgenic mouse line was generated to facilitate the study of hypothalamic GnRH neurons in which the green fluorescent protein (GFP) is genetically targeted to these cells (Suter et al. 2000a). The expression of GFP was detected in 84–94% of GnRH immune-positive neurons. This mouse line was used in studies aimed at electrophysiological characterization (Suter et al. 2000b; Pielecka-Fortuna et al. 2008; Christian et al. 2005; Farkas et al. 2010, 2016) and cell type-specific (or singe-cell) genomic analysis of the GnRH neurons (Vastagh et al. 2015, 2016).

#### The *Gcg*-*Cre* mouse line

To develop a better understanding of GCG neuron function, a transgenic mouse line expressing Cre recombinase from the *Gcg* locus of a bacterial artificial chromosome (BAC) was developed (Gaykema et al. 2017) (Department of Pharmacology, University of Virginia School of Medicine, Charlottesville, Virginia, USA). The line was rederived at the Medical Gene Technology Unit of the IEM, Hungary according to a protocol published earlier (Van Keuren and Saunders 2004). Briefly, fertilized eggs for embryo transfer were obtained by mating super-ovulated egg donor C57Bl6 females with *Gcg*-*Cre* males. Offspring of the F1 generation were genotyped with a real-time PCR system using primers Cre + 5′-GTGAAACAGCATTGCTGTCAC-3′, Cre—5′-TGCTTCTGTCCGTTTGCCGGT-3′. Males homozygous for the *Cre* transgene (*Cre*^+/+^) were used in further crossbreeding procedures.

#### The GCG-ChR2 mouse line

*Gcg-Cre* male mice were crossed with channelrhodopsin-2 (ChR2) reporter mice [Ai32(RCL-ChR2(H134R)/EYFP)] (Madisen et al. 2012) to characterize the expression pattern of the transgene. Offspring were genotyped for both *Cre* and enhanced yellow fluorescent protein (*eyfp)* transgenes to measure the copy number of the transgenes using YFP reference gene assay mix. Animals homozygous for *Cre* and *eyfp* were selected for morphological experiments, as well as for crossbreeding with the GnRH-GFP mouse line. The  GCG neuron-specific expression of ChR2 was validated with double immunofluorescence against GLP-1 peptide and eYFP.

#### The GCG-ChR2-GnRH-GFP mouse line

The triple-transgenic, GCG-ChR2-GnRH-GFP mouse line was established for the purpose of an optogenetic study using in vitro slice electrophysiology. In this experimental design, blue laser light stimulation of the ChR2-expressing axons of GCG neurons distributed in the medial preoptic area and whole cell patch recording from GFP-expressing GnRH neurons were performed simultaneously. To accomplish this goal, homozygous male GCG-ChR2 mice were crossed with female GnRH-GFP mice. Presence of GnRH-GFP transgene in the offspring of the first generation was validated using real-time qPCR: the relative copy number of the *gfp* transgene (3 vs 2) was still distinguishable using this method.

### Double and triple label fluorescent immunocytochemistry

Adult, double and triple transgenic mice were deeply anesthetized with a mixture of ketamine (25 mg/kg body weight) and Xylavet (5 mg/kg body weight) injected intraperitoneally, then were perfused transcardially with 4% paraformaldehyde (pH = 7.6, in PBS) for 10 min at a flow rate of 4 ml/min. Brains were removed rapidly from the skulls and post-fixed in the same fixative overnight at 4 °C. On the next day, brains were sectioned in the coronal plane at 25 µm thickness using a Vibratome (Leica Inc, Vetzlar, Germany). Sections containing the medial preoptic area (mPOA) and the nucleus tractus solitarii (NTS) were processed for heat antigen retrieval in 0.01 M sodium-citrate buffer (pH 6.0) at 80 °C for 30 min, then were pre-treated with 0.5% H_2_O_2_ and permeabilized with 0.5% Triton X-100 for 20 min. The following primary antisera were used: anti-GnRH [raised in guinea pig #1018, diluted in 1:10.000 (gift from E. Hrabovszky)], anti-GFP (raised in goat, Abcam #5450, diluted in 1:10.000, commercially available) and anti GLP-1 (raised in rabbit, Peninsula Labs #T-4363, diluted in 1:5000) for 18 h. Secondary antisera (Alexa 488 conjugated anti guinea-pig IgG; Cy3 conjugated anti-goat IgG, biotin conjugated anti-rabbit IgG; [Jackson ImmunoResearch Europe Ltd, UK]) were all diluted at 1:500 in serum diluent (2% NHS in PBS). Cy5 conjugated streptavidin was applied in 1:1000 (Jackson).

### Tissue processing for 3DISCO-based double fluorescent immunocytochemistry

In this study, the 3DISCO protocol (Erturk et al. 2012) was applied with minor modifications.

Animals were handled and perfused as described above. Brains were either sliced at 1 mm placed in mouse brain matrix or sectioned at 500 um thickness using a vibratome (Leica VT1000S, Leica Biosystems, Wetzlar, Germany) in coronal and sagittal planes (Supplementary Fig. 1). Slices were rinsed several times in PBS, then were permeabilized with PBS-GT (PBS containing 0.5% Triton-X100 in PBS containing 0.1% gelatin (GT) and 0.05% merthiolate) for 3 days at room temperature (RT) under constant agitation. For fluorescent immunocytochemical double labeling, brain slices were incubated with the primary antibodies [anti-GLP-1 (rabbit; #T-4057 Peninsula Labs, 1:2000) and anti-GnRH (guinea-pig; 1:5000)] diluted in PBS-GT, for 7 days at RT. Several washes (6 × 60 min) in PBS-GT were followed by the incubation with secondary antibodies (1:500; Cy3 anti-rabbit IgG and Cy5 anti-guinea pig IgG; Jackson ImmunoResearch Europe Ltd., Cambridge House, UK) for 7 days at RT. Specificity of the primary antibodies were validated elsewhere (Farkas et al. 2016).

Slices were then rinsed in PBS-GT 6 × 60 min and then in PBS for 30 min. For dehydration and lipid removal steps, series of increasing tetrahydrofuran (THF, Sigma-Aldrich) were used (50% THF overnight at 4 °C, 80% and 3 × 100% THF for 30 min at RT). For optical clearing, slices were immersed in dibenzyl-ether (DBE, Sigma-Aldrich) for at least 15 min, then were kept either in DBE for storage, or covered using a glass slide with custom-made plastic supporting frame adjusted for the actual specimen thickness. The specimen was cover-slipped with DBE.

### Evaluation of double-labelled specimens

Stack of images was acquired with a LSM780 laser scanning confocal system equipped with an Axio Imager 2 microscope (Carl Zeiss Microscopy GmbH, Jena, Germany) at 10x (Plan-Apochromat 10x/0.45) 20x (Plan-Apochromat 20x/NA 0.8) or 40x (LD C-Apochromat 40x/1.1 W) objective magnifications. To maximize the emitted signal intensity, sequential scanning mode was set in the software (ZEN v. 2.3; Carl Zeiss). For Cy3-coupled labeling, an argon laser (514 nm), MBS 458/514 beam splitter and a 538—680 nm excitation filter were used. At the same time, to detect Cy5 emission, a HeNe laser (633 nm), MBS 488/561/633 beam splitter and 638–759 nm BP excitation filter were applied.

### Quantitative analysis, statistics

For quantitative analysis, the 20 × objective was used at 1024 × 1024-pixel *xy* image resolution. During scanning process, the pinhole diameter was set to 1 AU (Airy unit) in the acquisition software. The size of the optical thickness was 2 µm. The optical sectioning was adjusted to match pinhole (z-step interval 0.98 µm). The scanning area was positioned over the medial septum-diagonal band of Broca-medial preoptic area where the majority of hypophysiotropic GnRH neurons resides.

All GnRH-IR perikarya were counted in the z-stacks of scanned areas. GnRH-IR neurons receiving GLP-1-IR axonal appositions were also counted using the orthogonal view of the z-stacks. The ratio of GnRH-IR perikarya targeted by GLP-1 axons was calculated. The ratio of all GnRH-IR dendrites and those contacted by GLP-1-IR axons was estimated similarly. Student’s *t*-test was used to test the significance of the differences in the morphological data and considered as significant at *p* < 0.05.

### Acute brain slice preparation for electrophysiology

Triple-transgenic (GCG-ChR2-GnRH-GFP) adult, male mice were deeply anesthetized using Isoflurane inhalation. The brain was removed rapidly and immersed in ice cold low-sodium artificial cerebrospinal fluid (low-Na aCSF) bubbled with a mixture of 95% O_2_ and 5% CO_2_. The solution contained the following (in mM): saccharose 205, KCl 2.5, NaHCO_3_ 26, MgCl_2_ 5, NaH_2_PO_4_ 1.25, CaCl_2_ 1, glucose 10. Parasagittal acute brain slices of 250 μm thickness containing GnRH-GFP neurons next to the median plane were prepared with a Leica VT-1000S vibratome (Leica Microsystems, Wetzlar, Germany) in the ice-cold oxygenated low-Na aCSF. The slices were equilibrated in normal aCSF (in mM): NaCl 130, KCl 3.5, NaHCO_3_ 26, MgSO_4_ 1.2, NaH_2_PO_4_ 1.25, CaCl_2_ 2.5, glucose 10, saturated with O_2_/CO_2_ for 1 h. Initial temperature of aCSF was 33 °C which was left to cool to room temperature during equilibration. Recordings were carried out in oxygenated aCSF at 33 °C. Axopatch-200B patch-clamp amplifier, Digidata-1322A data acquisition system, and pCLAMP 10.4 software (Molecular Devices Co., Silicon Valley, California, US) were used for recording. Cells were visualized with a BX51WI IR-DIC microscope (Olympus Co., Tokyo, Japan) located on a S’Table antivibration table (Supertech Inc., Hungary-Switzerland). The patch electrodes (OD = 1.5 mm, thin wall, WPI Inc., Sarasota, FL) were pulled with a Flaming-Brown P-97 puller (Sutter Instrument Co., Novato, CA, US). The head-stage of the amplifier was fitted to an MHW-3 micromanipulator (Narishige Inc., Tokyo, Japan). Hypothalamic GnRH-GFP neurons were identified by brief illumination at 470 nm using an epifluorescent filter set, based on their green fluorescence, typical fusiform shape and characteristic topography.

### Whole-cell patch-clamp experiments, optogenetic stimulation, data analysis

Exit of the glass fiber of the 473 nm emission wavelength IKE-473-100-OP laser (IkeCool Inc., Los Angeles, California, US) was set onto the surface of the brain slice. Then a GnRH-GFP neuron was patch clamped in the close vicinity (in 200–300 µm) of the end of the glass fiber. Whole-cell patch clamp measurements were carried out to record postsynaptic currents (PSCs) in voltage-clamp or action potentials (APs) in current-clamp mode. Pipette resistance was 1–2 MΩ, resistance of giga-seal was 2–3 GΩ. The pipette solution contained (in mM): HEPES 10, KCl 140, EGTA 5, CaCl_2_ 0.1, Mg-ATP 4, Na-GTP 0.4 (pH = 7.3 with NaOH). Osmolarity was adjusted to 295–300 mOsm with sorbitol. Whole-cell patch clamp measurements were carried out to record PSCs and APs. The neurons were voltage clamped at a pipette holding potential − 70 mV (for PSCs) and current clamped at 0 pA (for APs). Duration of a laser pulse was 10 ms with 3 mW power.

Low-frequency train of laser pulses was applied at 0.2 Hz (60 pulses totally) for PSCs and then records of the PSC responses to 60 pulses were averaged (10 neurons from 5 mice).

Measurement of the firing rate was carried out at higher laser shooting frequency (5–40 Hz, 10 ms pulse width, 3 mW laser power), starting with a 30 s long recording with no laser (for control purposes) and with a subsequent 30 s long laser shooting period. Analysis of firing rate change was carried out on the percentage data calculated by dividing the firing rate of the second half minute period with that of the first one, on 10 recorded cells from 5 animals. After the recording, the GLP-1 receptor (GLP-1R) antagonist Exendin-3(9-39) (1 μM) was added to the aCSF and 10 min later the measurement was repeated in the presence of the antagonist.

Recordings were stored and analyzed off-line using the Clampfit module of the PClamp 10.4 software (Molecular Devices Co.). Statistical significance was analyzed by Student’s *t*-test on the percentage data and considered as significant at *p* < 0.05.

## Results

## Networking of the central GLP-1 neuron system with GnRH neurons of mice: neuroanatomical evidence

### Characterization of the GCG-ChR2 mouse line

#### Rederivation and genotyping of the *Gcg*-*Cre* mice

The performed real-time PCR tests verified the expected copy number of the *cre* transgene after selective crossing of heterozygous (*Cre/* +) parents from the F1 generation of the rederived gcg-cre mouse line (Supplementary Table 1). Only mice homozygous for the transgene (*Cre*^+/+^) were used in further crossbreeding processes to create double and triple transgenic lines.

#### Geno-and phenotyping of the GCG-ChR2 mouse line

**Fig. 1:**
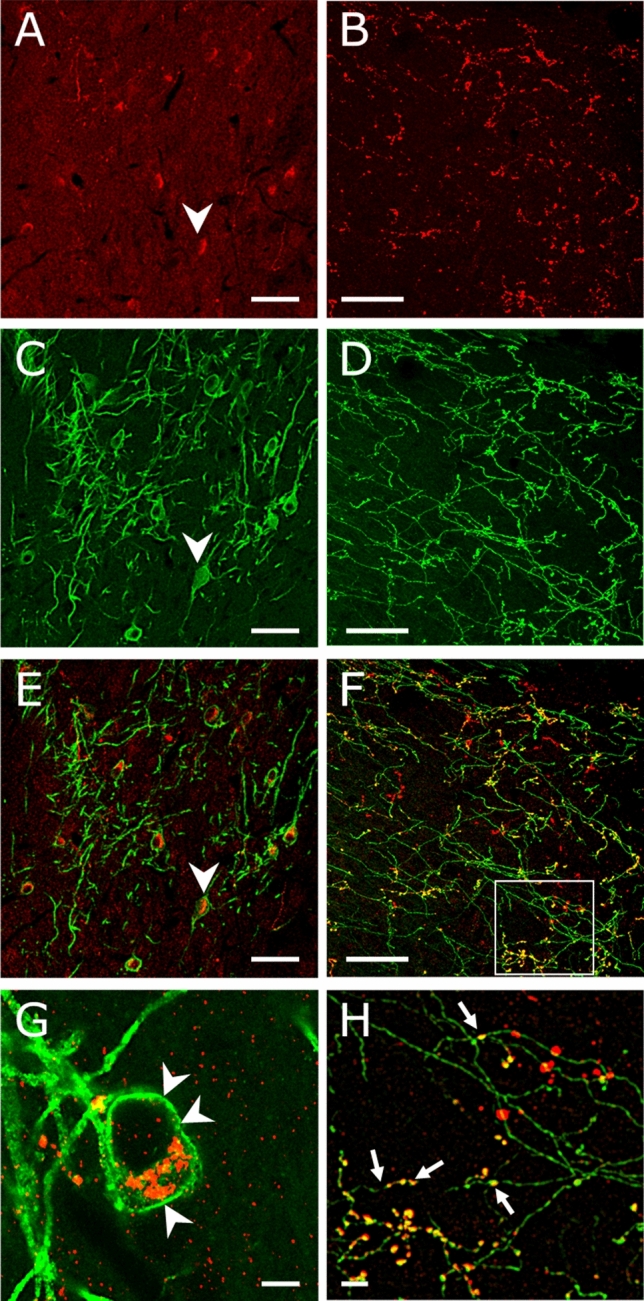
**Colocalization of GLP-1 and ChR2-eYFP in GCG neurons and their projecting axons.** Co-expression of GLP-1 (red) and ChR2-eYFP (green) in neuronal perikarya of the nucleus tractus solitarii (NTS) (A, C, E, arrowheads) and axons in the medial preoptic area (mPOA) (B, D, F) revealed by immunofluorescence. (G) GLP-1 peptide is localized in the cytoplasm (red) of ChR2-eYFP neuron of the NTS whose cell membrane expresses channelrhodopsin (green, arrowheads). Area delineated on F (white rectangle) is represented on H, at a higher power of magnification. (H) GLP-1 and eYFP colocalize in axon varicosities (yellow, arrows) within the MPOA. Scale bar: 50 µm (A-F), 5 µm (G, H)

Homozygous  *Gcg*-*Cre* male mice were crossbred with Ai32 reporter female mice through several generations to get double homozygous male and female offspring and maintain a new mouse line for morphological characterization of the GCG-cre mouse. Double fluorescent immunocytochemistry revealed that the GLP-1 peptide colocalizes with the ChR2-eYFP fusion protein in the territory of the nucleus tractus solitarii (NTS) (Fig. 1a, c, e) showing no ectopic expression in this region which indicates a specific ChR2-eGFP ‘tagging’ of the GCG neurons in the NTS. The GLP-1 peptide accumulated in the perinuclear cytoplasm, while the fusion protein ChR2-eYFP was localized in plasma membrane. (Fig. 1g). In the medial preoptic area (mPOA), ChR2-eYFP and GLP-1 immunoreactivity overlapped in axons (Fig. 1b, d, f). An uneven distribution pattern of the two immunofluorescent signals within the same axonal profile was observed. While ChR2-eYFP showed a continuous expression in the axolemma, GLP-1 peptide was more prominent in axon varicosities (Fig. 1h).

### 3DISCO-based double immunocytochemistry reveals the innervation of a subset of GnRH neurons by GLP-1 axons in the mouse forebrain

**Fig. 2:**
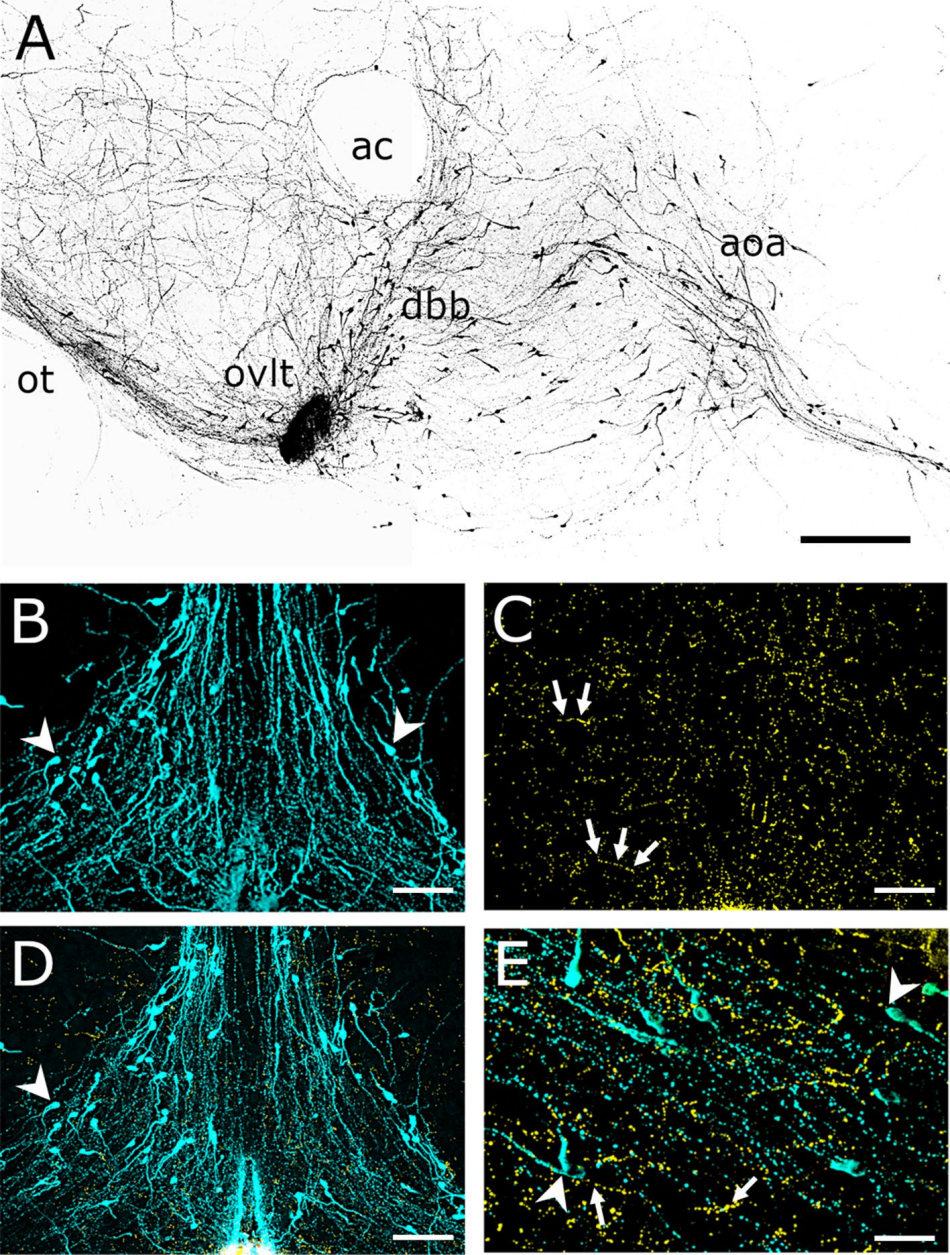
**Immunofluorescent detection of GnRH and GLP1 immunoreactivities by confocal z-stack imaging method in thick, 3DISCO sections of the mouse brain.** A characteristic distribution of GnRH neurons in a 1 mm thick, paramedian-sagittal slice. The 3D reconstruction was generated from 185 confocal images, 1 micrometer thick each. Distribution of GnRH neurons (arrowheads, B) and GLP-1 immunoreactive axons (arrows, C) in the same 500 micrometer thick coronal section. (D) Merged image of B and C. E: 3D reconstruction of GnRH cells and GLP-1 axons in a 60 µm thick image stack. Scalebar: 500 µm (A), 50 µm (B-D), 20 µm (E). ac: anterior commissure; aoa: anterior olfactory area; dbb: diagonal band of Broca; oc: optic tract; ovlt: organum vasculosum laminae terminals

**Fig. 3:**
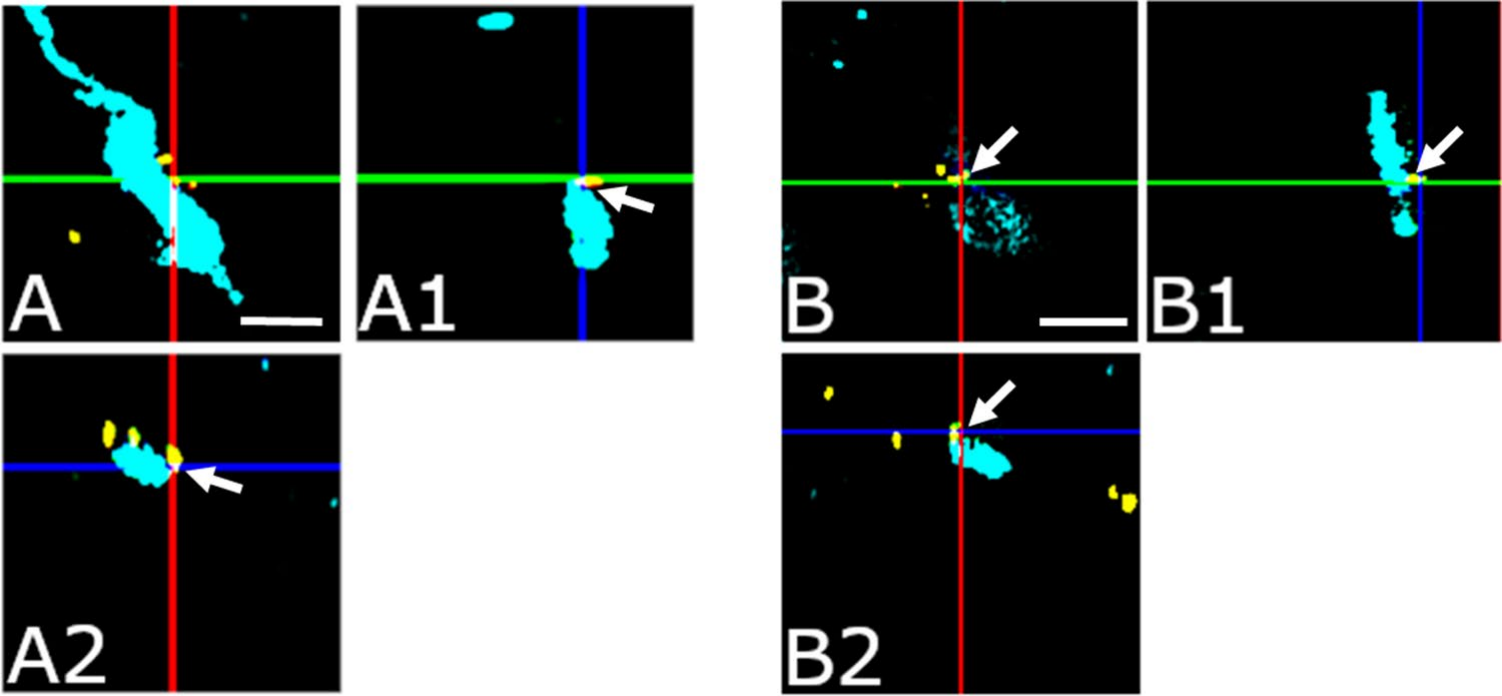
**Innervation of GnRH neurons by GLP-1 immunoreactive axons shown in orthogonal views.** Arrowheads point to communication sites between GLP-1 axons (gold) and GnRH neurons (turquoise). The orthogonal views indicate the juxtaposition of GLP-1 axons to the cell bodies (A; A1: xz and A2 in the yz plane) and dendrite (B; B1: xz, B2: yz plane) of GnRH neurons. Scale bar: 5 µm

**Fig. 4:**
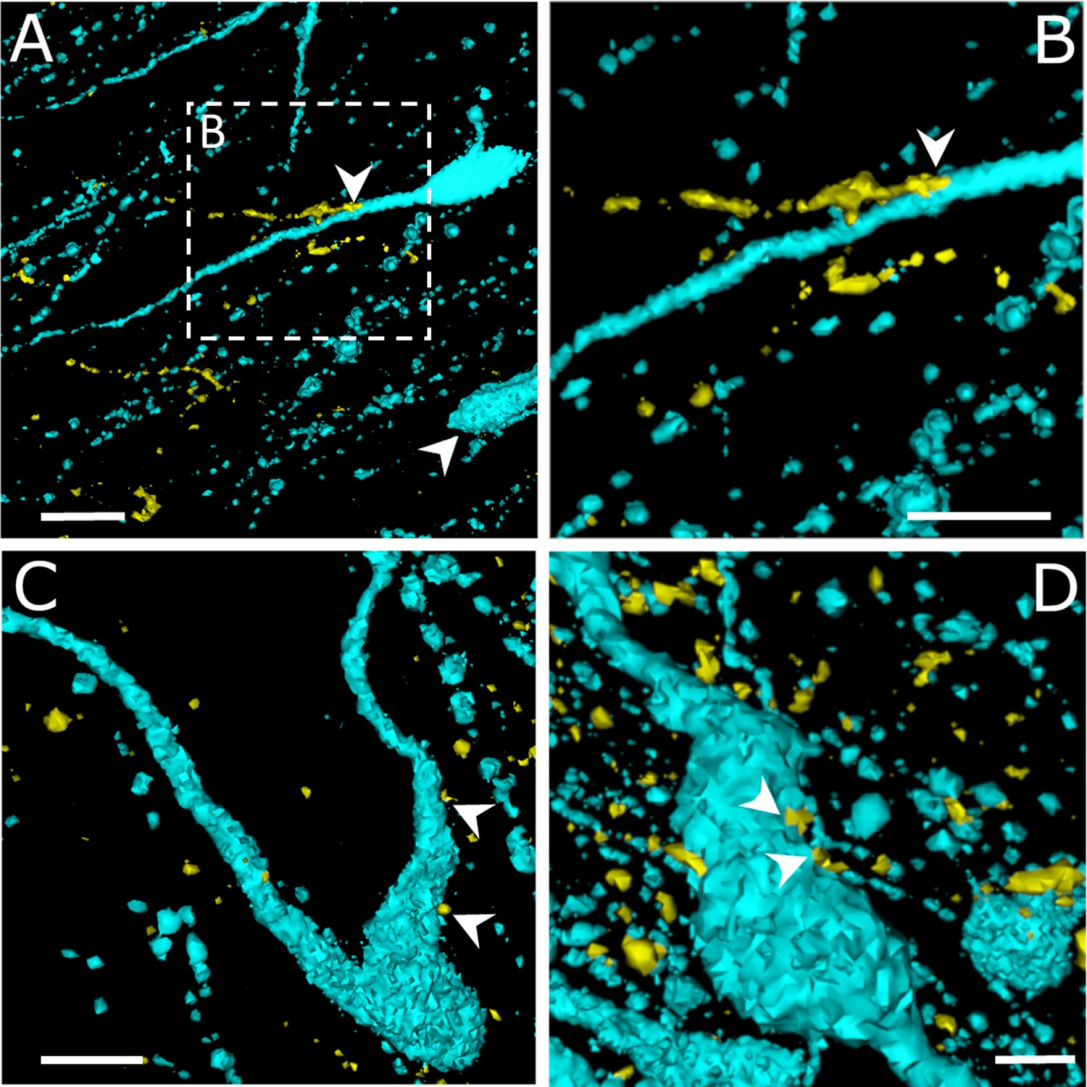
**High power visualization of communication/contact sites between the GLP-1 and GnRH neuronal systems by 3D reconstruction of confocal image stacks.** The reconstruction was performed by the ImageJ 3D Viewer plug-in. Arrowheads point to juxtapositions of GLP-1 axons to dendrites (A, B, C) and perikaryon of (D) of GnRH neurons. GnRH cells receive both single (A, B) and multiple (C, D) GLP-1 axonal inputs. Scale bars: 10 µm (A, B) 5 µm (C), 2 µm (D)

The 3DISCO-based immunocytochemistry ensured a novel approach to visualize and analyze a thick tissue block (500–1000 µm) compared to traditional immunocytochemical methods. With the aid of this protocol, the characteristic rostro-caudal distribution pattern of the GnRH neurons and the arborization of their processes were demonstrated by reconstructing series of optical slices (Fig. 2a). It was also verified that GnRH neurons and GLP-1-IR axons were distributed in the same territory of the OVLT-mPOA in z-projected image stacks (Fig. 2b–e). The GLP-1-IR axons formed bouton-like appositions on the surface of the GnRH neurons. The orthogonal view analysis of the stacks made possible the identification of individual contacts on the surface of cell bodies (Fig. 3a–a2) and dendrites (Fig. 3b–b2) of GnRH neurons. 3D reconstruction of image stacks revealed that GnRH neurons may receive single (Fig. 4a, b) and multiple (Fig. 4c, d) contacts by GLP-1 axons both on their dendrites and somata.

### Quantification of the GLP-1-IR neuronal input onto GnRH neurons

**Fig. 5:**
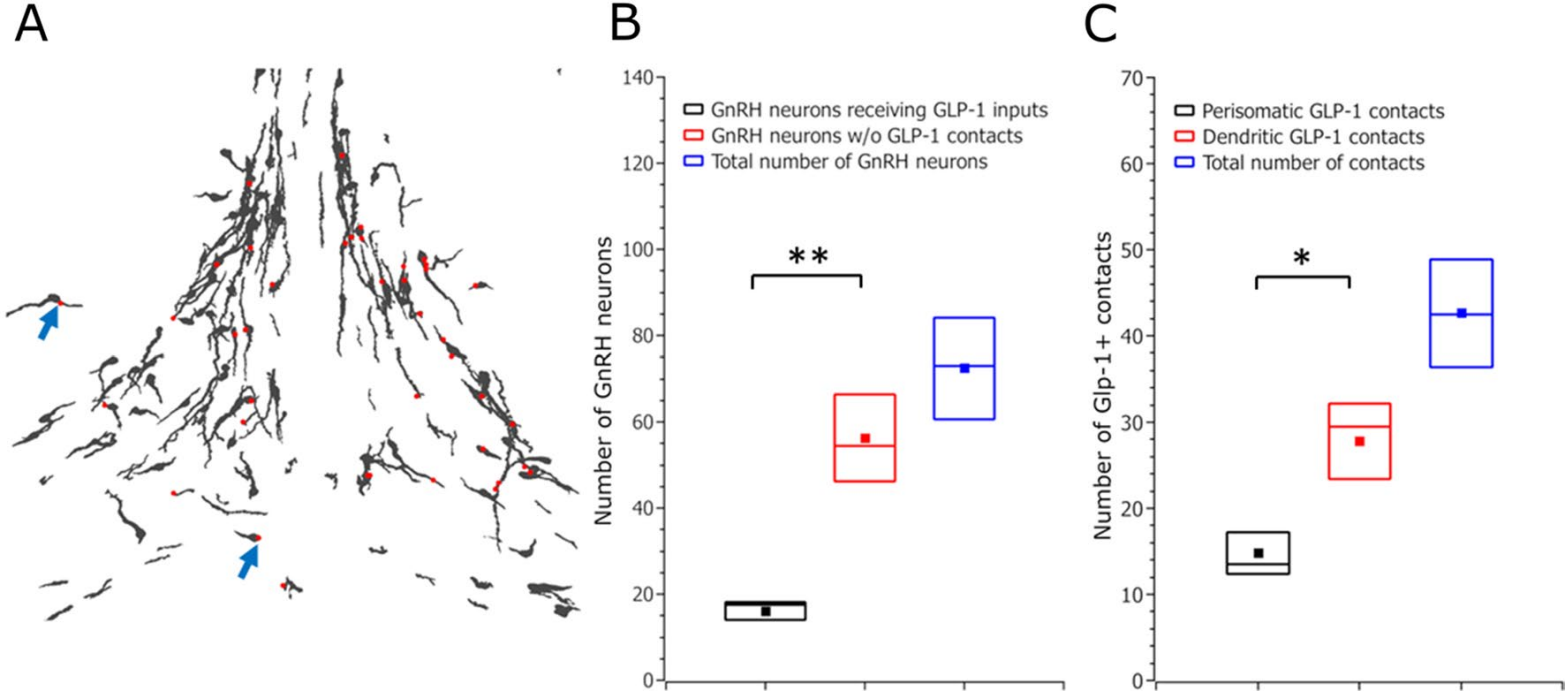
**Quantitative analysis of appositions established by GLP-1-IR axons and GnRH neurons.** A. The scheme shows an example of the distribution pattern and location of GLP-1 boutons (red, arrows) apposing GnRH somata and dendrites (black) in the territory of mPOA. B. Only a subset of GnRH neurons is targeted by GLP-1 axons (*: p < 0.05). C. The number of the contacts that target GnRH immunoreactive dendrites is significantly higher compared to perisomatic appositions (**: p < 0.005). The color-coded rectangles represent means ± SEM. The horizontal lines inside the rectangles indicate medians of the data

For quantification, 435 GnRH neurons and 256 GLP-1-IR contacts were identified and counted in orthogonal view of image stacks (Fig. 5a, schematic illustration). Only a sub-population of GnRH neurons received GLP-1 axons: the mean was 16 ± 2 innervated vs 56 ± 10 non-innervated GnRH neurons number per animal (97 vs 338 in total, respectively) which was significant across the samples (Student’s *t*-test, two tailed, *p* = 0.003) (Fig. 5b). The ratio of GnRH neurons that received GLP-1-IR contacts vs. all GnRH neurons was 0.23 ± 0.014 s.e.m., showing that 23 percent of the GnRH neurons were contacted by GLP-1 axons.

The majority of the GLP-1 axon profiles were found on the dendritic processes of the GnRH neurons compared to the perikarya: 15 ± 2 (mean ± SEM) GLP-1 profiles were found on GnRH perikarya and 28 ± 4 (mean ± SEM) appositions were received by GnRH dendrites, per animal (89 vs. 167 in total, respectively), which showed significant difference (Student’s *t*-test, two tailed, *p* = 0.026) (Fig. 5c).

## GLP-1 axons modulate the electrophysiological activity of GnRH neurons: optogenetic evidence

### Characterization of the triple transgenic mouse strain used for optogenetics

**Fig. 6:**
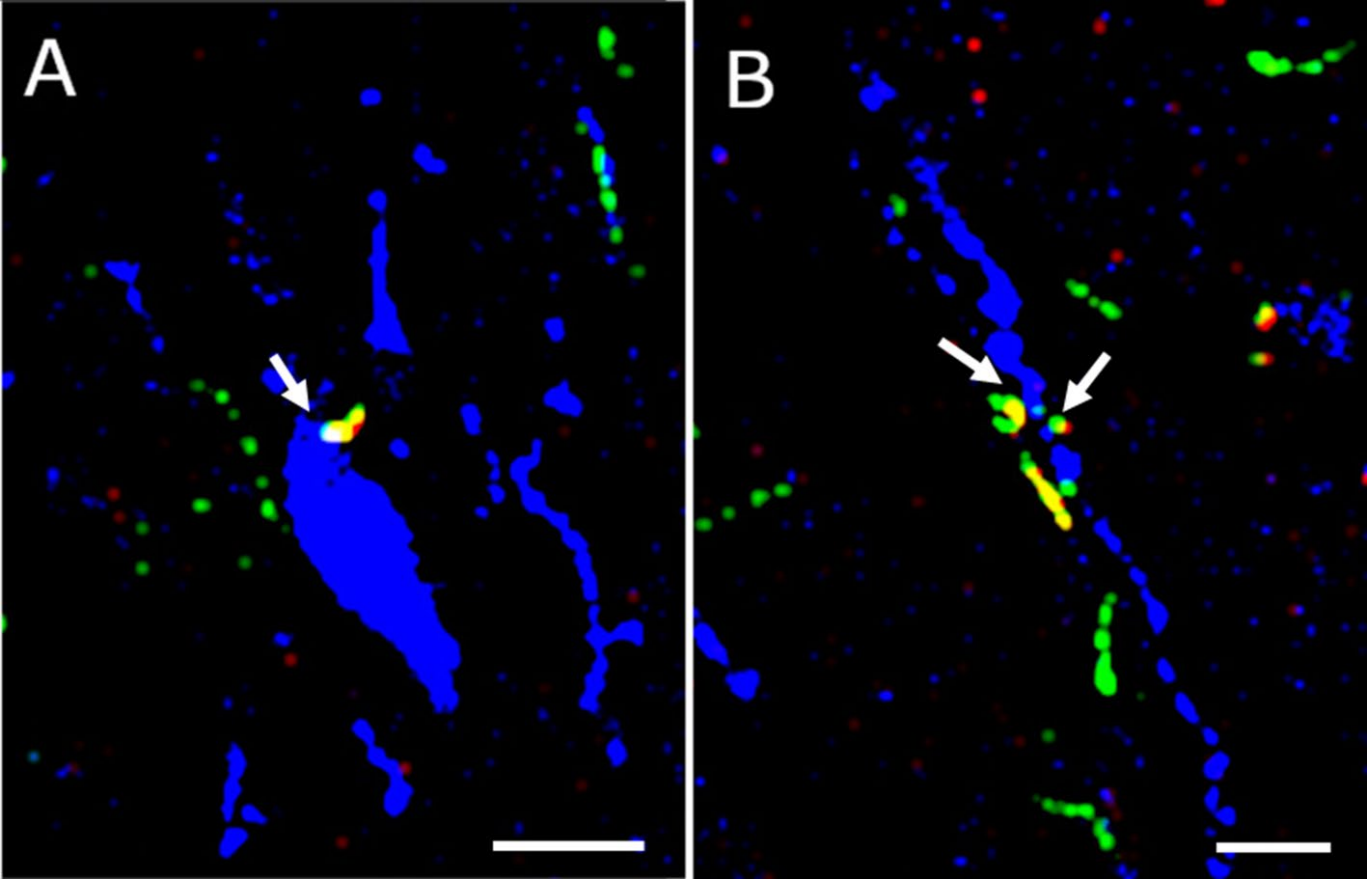
**GLP-1 and ChR2-eYFP co-localize in axons juxtaposed to GnRH neurons.** Triple immunofluorescent labeling shows co-expression (yellow) of GLP-1 peptide (red) and channelrhodopsin-fused eYGP (green) in axon-beads juxtaposed to GnRH-immunoreactive (blue) perikaryon (A, arrow) and dendrite (B, arrow). Scale bar: 5 µm

The double homozygous GCG-ChR2 male mice were crossed with homozygous GnRH-GFP females to get triple transgenic GCG-ChR2-GnRH-GFP mice (heterozygous for the transgenes) to be used primarily for optogenetic investigations. The triple-transgenic mouse line was characterized by fluorescent immunocytochemistry. Triple (anti-GnRH, anti-GFP and anti-GLP-1) staining of the mPOA revealed that both the ChR2-eYFP fusion protein (GFP) and the GLP-1 peptide co-localize in axonal projections innervating the mPOA. Furthermore, the double labeled axon beads were juxtaposed to perikarya (Fig. 6a) and dendrites (Fig. 6b) of GnRH neurons. This finding confirms the feasibility of using the triple transgenic mouse line in further studies by optogenetics.

### Effects of optogenetic stimulation of GCG-ChR2 axons upon the activity of GnRH-GFP neurons

**Fig. 7:**
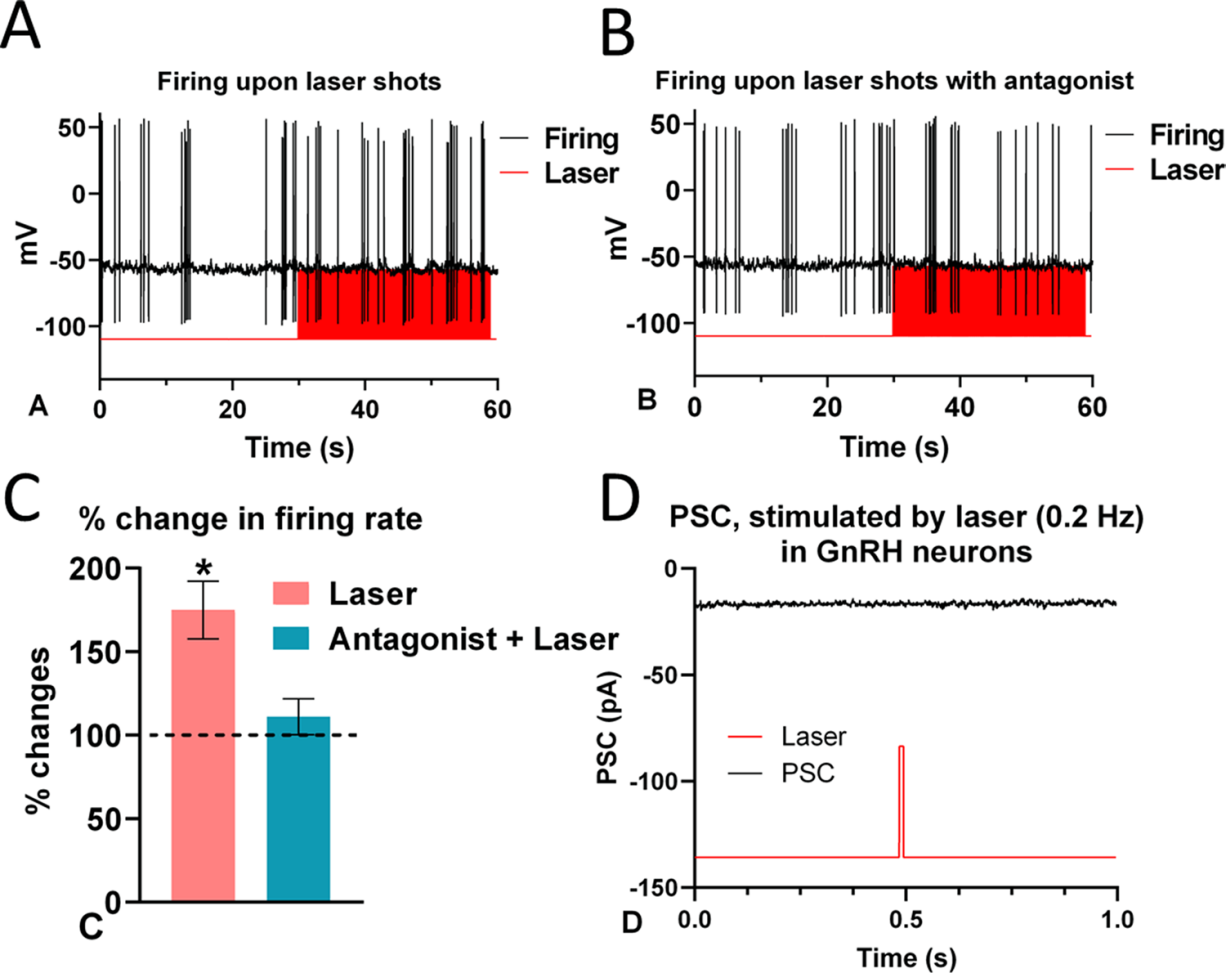
**Effect of the high frequency (20 Hz) laser stimulation on firing of GnRH neurons.** Representative AP recordings in untreated (A) and GLP-1 receptor antagonist, Exendin-3(9-39) (1 μM) pretreated (B) brain slices prepared from the basal forebrain of the used triple transgenic mouse. Bar graph shows a significant change in the firing rate (C). The graph also demonstrates the elimination of the effect upon pretreatment the brain slice with the GLP-1R antagonist. PSCs could not be evoked by laser shots (D). *: p < 0.05. The duration of laser stimulation is marked by red bars in (A), (B) and (D)

High frequency laser stimulation (20 Hz) increased the firing rate of GnRH neurons (in 6/10 neurons), from 0.47 ± 0.07 Hz of the control period to its 175 ± 17.3% (*p* = 0.0007, Student’s *t*-test) (Fig. 7a, c) with no alteration in various parameters shaping the APs (Table 1). Slightly weaker response was recorded at 10 Hz, whereas higher (40 Hz), or lower (5 Hz) laser frequencies had no effect on the firing rate (not shown). The facilitatory effect of laser stimulation upon the firing activity of GnRH neurons was abolished by GLP-1 receptor antagonist (111 ± 10.7%, *p* = 0.3236) (Fig. 7b, c).

**Table 1:** Various parameters of the APs, before and during the laser  stimulation. The Student’s *t*-test was applied to the percentage data. Df = degree of freedom; p = probability; t = “t” value of Student’s *t*-test; AP = action potential; AHP = after hyperpolarization

df = 5	Control (*n* = 6)	Laser activated (*n* = 6)	*t*	*p*
AP amplitude	108.8 ± 5.40 mV	96.3 ± 3.02%	1.225	0.2751
Time to peak of AP	1.89 ± 0.209 ms	92.1 ± 8.25%	0.9576	0.3823
AHP amplitude	− 39.65 ± 1.542 mV	99.0 ± 2.58%	0.3876	0.7143
Time to AHP peak	3.71 ± 0.253 ms	96.2 ± 4.18%	0.9091	0.4050
Half-width	1.07 ± 0.062 ms	103.8 ± 4.06%	0.9360	0.3923
Time to half-amplitude rise	1.33 ± 0.156 ms	89.5 ± 9.12%	1.151	0.3017
Time to decay half-amplitude	2.40 ± 0.139 ms	95.8 ± 10.13%	0.4146	0.6956
Rise tau	0.66 ± 0.336 ms	112.1 ± 9.62%	1.258	0.2640
Decay tau	5.88 ± 7.303 ms	128.4 ± 17.04%	1.667	0.1565

In contrast, low frequency laser stimulation (0.2 Hz, average of 60 laser shots) did not result in any detectable laser-triggered PSCs in GnRH neurons (Fig. 7d).

A linear regression analysis was carried out to examine whether change in firing rate depends on the basal activity. The value of the Goodness-of-fit (*r*^2^) parameter is near zero (*r*^2^ = 0.03478) suggesting that change in firing rate is independent of the basal activity. Repeated stimulations each followed by a 1 min silent period were also carried out. The measurements demonstrated that the firing pattern returned to its unstimulated level after the pause and the subsequent stimulation also resulted in an increase of firing rate.

## Discussion

Earlier studies have shown that GLP-1 can control the HPG axis centrally via interaction with the GnRH system (Beak et al. 1998; Farkas et al. 2016). Furthermore, its key regulator, the hypothalamic kisspeptin neuron circuit is also influenced by GLP-1 (Oride et al. 2017; Heppner et al. 2017). In the present study, we have provided compelling evidence for the modulatory role of the brain-derived GLP-1 upon GnRH neurons. Accordingly, (1) GLP-1-axons arising from the NTS innervate a subpopulation of hypophysiotropic GnRH neurons (23%) in the OVLT-mPOA area; (2) Mapping the surface of GnRH neurons for juxtaposed GLP-1 axons in orthogonal view revealed that dendrites of GnRH neurons receive almost twice as many GLP-1-IR boutons than cell bodies; (3) Optogenetic stimulation of axons projecting from GCG-ChR2-expressing neurons of the NTS to the OVLT-mPOA area, the main residence of the hypophysiotropic GnRH neurons, significantly increases the firing rate of GnRH cells and (4) blocking the GLP-1R by Exendin 3 prior to the optogenetic activation prevents the increment in firing of GnRH cells, indicating that GLP-1 released from the photo-stimulated axons is responsible for the facilitatory action.

## Networking of the central GLP-1 neuronal system with GnRH neurons

### Quantification of the neuronal GLP-1 input to hypophysiotropic GnRH cells

GLP-1-synthesizing neurons of the medulla oblongata project to several areas in the rodent brain, including the hypothalamus where GLP-1 acts as a neuromodulator in a variety of neural circuits known to regulate feeding, appetite and body weight (Liu et al. 2017; Lopez-Ferreras et al. 2018; Burmeister et al. 2017; Katsurada et al. 2014; Renner et al. 2012), stress and adaptation (Herman 2018; Ghosal et al. 2017; Holt et al. 2019), food and drug reward (Skibicka 2013) and reproduction (Beak et al. 1998; MacLusky et al. 2000; Outeirino-Iglesias et al. 2015). Accordingly, the G-protein-coupled receptor of the GLP-1 peptide is expressed in certain hypothalamic nuclei and also in the preoptic area where hypophysiotropic GnRH neurons reside (Cork et al. 2015; Merchenthaler et al. 1999; Trapp and Cork 2015; Farkas et al. 2016; Goke et al. 1995; Graham et al. 2020; Jensen et al. 2018). The NTS and the reticular nucleus of the medulla give rise to the ascending axon projections that innervate the aforementioned brain sites and provide the neuromodulator substance, GLP-1 for communication (Holt et al. 2019; Katsurada et al. 2014). The medial preoptic area of the rodent brain is rich in GLP-1-IR axons (Larsen et al. 1997) and the region also expresses GLP-1R (Cork et al. 2015; Graham et al. 2020). Our present immunocytochemical results are in line with these observations, furthermore, they identify the GnRH neuron population, as one of the targets of the GLP-1 input in this region. Double and triple fluorescent labelling experiments revealed that GLP-1-IR axons provide neuronal inputs to GnRH cells. Based on this methodology, the abundance of GLP-1-IR appositions on GnRH neurons was measured. The results indicate that GnRH neurons are only partially innervated (23%) by GLP-1-IR axons. These neurons are most frequently located in the vicinity of the OVLT and the medial preoptic nucleus, regions that host hypophysiotropic GnRH cells. The distribution of the GLP-1-IR boutons is disproportionate along the GnRH neuron surface showing a significantly heavier innervation pattern of the dendrites (65%) than the somata (35%). This is in agreement with earlier studies, where neuronal inputs to dendritic profiles were found significantly higher than to perikarya in male mice (Silverman et al. 1988; Moore et al. 2018a; Campbell et al. 2005). Hypophysiotropic GnRH neurons have rather long dendritic processes which receive glutamatergic and GABAergic synaptic inputs abundantly (Moore et al. 2018a). The regulation/modulation of GnRH neurons by neuropeptides contributes to the success of reproduction (Spergel 2019). Kisspeptin (KP) is a potent activator of GnRH neurons (Clarkson et al. 2010; Zhang et al. 2008; Irwig et al. 2004). In the rostral preoptic area of male mouse, 10% of GnRH neurons are innervated by KP axons, whereas 40% of the cells receive KP inputs in the same territory of the female brain (Clarkson and Herbison 2006). We have previously shown that a higher portion, about 80% of GnRH neurons, were contacted by KP axons in OVX and OVX + E2 supplemented animals, and the average number of KP boutons per GnRH neuron was about two (Kallo et al. 2012). Regarding some other quantified, peptidergic inputs, a considerable portion of GnRH neurons (28%) also receives contacts by VIP-IR fibers in the mouse (Ward et al. 2009) and VIP increases the firing of 58–80% of these cells (Piet et al. 2016). RFamide-related peptide-3 (RFRP-3) containing boutons innervate 26% of GnRH neurons in male and diestrous female mice while inhibiting 41% of those cells (Ducret et al. 2009). Our present data resemble a similar innervation pattern by GLP-1 axons (23%) and also an electrophysiological response rate (60%) of GnRH neurons under optogenetic stimulation of the innervating GLP-1 axons.

The more intense innervation of dendrites than perikarya of GnRH neurons by GLP-1 axons is in line with previous studies. It has previously been shown that proximal segments of GnRH dendrites have a pivotal role in the physiology of the cells. They express active ion conductances, generate action potentials and contribute—via synaptic inputs—to the surge release of the hormone (Iremonger and Herbison 2015; Wang et al. 2020; Norberg et al. 2013).

### The feasibility of the 3DISCO method-based immunocytochemistry for mapping of neuronal circuits and quantitative evaluation of their anatomical interaction

The GnRH system of the rodent brain is rather unique. GnRH neurons derive from the olfactory placode, follow a long migratory path in the forebrain and establish a loose, scattered organization at the final destination sites (Forni and Wray 2015; Merchenthaler et al. 1984). These features make the anatomical mapping and sampling for molecular and electrophysiological purposes difficult. Regarding the mapping and reconstruction of identified neuronal systems in the brain, the whole mount fluorescent immunocytochemistry in combination with novel optical clearing techniques and the recently invented light-sheet microscopy have revolutionized the field (Vigouroux et al. 2017; Belle et al. 2014; Renier et al. 2014; Erturk et al. 2012; Zheng and Rinaman 2016).

To accomplish our goal, the simultaneous visualization of GnRH neurons and their GLP-1-IR axonal afferents in thick, whole mount preparations, the 3DISCO technique (Erturk et al. 2012) was combined with immunofluorescent staining (Belle et al. 2014). This approach allowed a large-scale sampling of the target, the GnRH neuron population and the three-dimensional (3D) morphometric analysis of the interacting GLP-1 and GnRH systems. GnRH neurons receiving GLP-1 immunoreactive boutons were counted in the 3D-reconstructed models and appositions were analyzed in orthogonal views. The relative high thickness of the tissue blocks (500–1000 µm) cut either in the frontal or sagittal planes ensured the proper sampling and counting of scattered GnRH neurons and also the estimation of GLP-1-IR axons terminating on the somata and dendrites of GnRH neurons. This approach provided a high throughput count of innervated neurons and the distribution of GLP-1 immunoreactive contacts due to the lack of distortion effects that may often come from processing series of thin sections. Apart from the advantages of the 3DISCO method, it also has some drawbacks (Vigouroux et al. 2017). Of note, tissue shrinkage during dehydration and clearing process can be a major limiting factor in the high-resolution imaging. In the present study none of the known limitations interfered with the investigations.

Similar approaches have been successfully applied to the 3D imaging of the KNDY neurons of the mammalian brain (Moore et al. 2018b), tyrosine hydroxylase, vasopressin and oxytocin neurons of the postnatal mouse brain (Godefroy et al. 2017) and neurons controlling fertility in the human brain (Casoni et al. 2016).

## Optogenetically released GLP-1 alters the electrophysiological activity of GnRH neurons

An in vitro optogenetic approach was used to elucidate the functional role of GLP-1 released from ascending axons of GCG neurons of the NTS upon the activity of GnRH cells. Photo-stimulation of these channelrhodopsin-2-expressing axons at 20 Hz increased the firing rate of GnRH neurons. A significant proportion of the cells (60%) responded which is in line with the current morphometrical analysis showing that only a subpopulation of GnRH neurons (~ 23%) received GLP-1-IR innervation. The increment in firing rate was abolished by antagonizing the GLP-1 receptor with Exendin 3 providing evidence that the released, biologically effective compound indeed was GLP-1. The frequency of the laser stimulation was also crucial in the evoked response. Low (0.2–5 Hz) and high (40 Hz) frequencies were ineffective, whereas stimulation at 20 Hz significantly increased the firing rate of GnRH neurons. The frequency-dependent response has been supported in recent optogenetic studies where the specific photo-stimulation of kisspeptin neurons of the AVPV area at 5–10 Hz evoked kisspeptin release resulting in delayed activation of GnRH neurons (Liu et al. 2011) and generated substantial increments in LH secretion (Piet et al. 2018).

GLP-1 has also been shown to stimulate various neurons. It enhanced the frequency of the postsynaptic currents in hypothalamic orexin neurons of mice by modulating their presynaptic glutamatergic afferents (Acuna-Goycolea and van den Pol 2004). In hippocampal CA3 neurons, both the spontaneous inhibitory postsynaptic current (sIPSC) amplitude and frequency were increased by GLP-1 (Korol et al. 2015), predominantly, also by presynaptic mechanisms (Babateen et al. 2017). In a previous study, we have also shown that activation of GLP-1 receptors in GnRH neurons triggers downstream mechanisms that modify both the retrograde endocannabinoid and nitric oxide (NO) signaling pathways to presynaptic GABAergic boutons (Farkas et al. 2016). The suppression of the endocannabinoid and activation of the NO retrograde messenger systems resulted in augmented firing of GnRH neurons and increased frequency of miniature postsynaptic currents (mPSCs).

GLP-1 neurons of the NTS express vesicular glutamate transport-2 indicating the glutamatergic nature of this cell population (Zheng et al. 2015). Therefore, they might use both the synthesized peptide (GLP-1) and the transmitter (glutamate) for inter-neuronal synaptic communication. In case of kisspeptinergic afferents of GnRH neurons arising from the anteroventral-periventricular area (AVPV), the axon terminals also co-synthesize GABA and glutamate and the recruitment of the fast amino acid and the slow kisspeptin release is frequency dependent (Liu et al. 2011). Glutamate and GABA drive to GnRH neurons occurs at low frequency (2 Hz) stimulation, while higher frequency (10 Hz) activation is required for the release of kisspeptin. Our patch clamp recordings under low-frequency (0.2–1 Hz) optogenetic stimulation found no laser-induced PSCs in GnRH neurons indicating the lack of fast-glutamate neurotransmission, whereas high-frequency (20 Hz) laser shots evoked GLP-1 secretion from the axon terminals resulting in an increased firing rate of GnRH neurons. Because the low frequency stimulation did not result in any glutamate-driven alterations in GnRH neurons, we carried out control recordings in neurons of the hypothalamic paraventricular nucleus (PVN) in acute brain slices from the same mice. This experiment with low-frequency stimulation demonstrated the clear presence of the laser-induced PSCs in PVN neurons that were abolished by pretreating the slice with the glutamate receptor antagonist, kynurenic acid (2 mM, unpublished observation). This finding supports the double transmitter/peptide phenotype character of GLP-1 axons and excludes the possibility that the absence of laser-induced PSCs in GnRH neurons was due to technical causes. In male mice, the postsynaptic currents are predominantly driven by GABA in GnRH neurons (Farkas et al. 2010, 2016), whereas in the female the PSCs are under both GABA and glutamate regulation in a gonadal cycle phase/estrogen feedback-dependent manner (Farkas et al. 2018; Christian and Moenter 2008). In this experiment male mice were used whose GnRH neurons might be less responsive to the released glutamate than neurons of the paraventricular nucleus. Another explanation for the discrepancy might be a differential expression of the glutamate neurotransmission machinery in GLP-1 fibers projecting to the PVN and the MPOA. These assumptions require further clarifications.

## Physiological significance of GLP-1 signaling upon the GnRH system

Earlier experiments have shown the capability of GLP-1 to enhance the release of GnRH from immortalized GnRH neurons (GT1-7 cells) and evoke an increase of plasma luteinizing hormone (LH) concentration after its intracerebroventricular administration in male rats (Beak et al. 1998). The treatment of female rats with GLP-1 in the morning of proestrus has been reported to double the amplitude of the preovulatory LH surge, modify estradiol and progesterone levels during the gonadal cycle and enhance the number of Graafian follicles, corpora lutea and the litter size (Outeirino-Iglesias et al. 2015). Studies focusing on cellular targets and mechanisms of GLP-1 action upon the HPG axis have revealed that GLP-1 acts via GLP-1 receptor expressed in GnRH neurons, it exerts excitatory effects on GnRH cells and both nitric oxide (NO) and endocannabinoid retrograde pathways are involved in the mediation of the effect targeting the presynaptic excitatory GABA-ergic system (Farkas et al. 2016). The present results indicate that a substantial portion of GnRH neurons receives GLP-1 afferents from the medulla and the optogenetically released GLP-1 enhances the firing of GnRH neurons in vitro. It is also noteworthy that GLP-1 neurons express leptin receptors (Scott et al. 2011), therefore, they might relay leptin signaling-related information to GnRH neurons.

## Conclusion

In summary, we reported the networking of GLP-1 neurons of the medulla with GnRH neurons in the mouse brain and showed that optogenetic stimulation of GLP-1 axons evoked GLP-1 release in the preoptic area that, in turn, accelerated the firing of GnRH neurons. The findings suggest that the GLP-1 neuron system, in addition to controlling powerfully feeding behavior, it also contributes to the modulation of the neuroendocrine reproductive axis via the hypophysiotropic GnRH system.

## Electronic supplementary material

Below is the link to the electronic supplementary material.

Supplementary file1 (PDF 150 KB)
