# Supplementary material for: Networking of glucagon-like peptide-1 axons with GnRH neurons in the basal forebrain of male mice revealed by 3DISCO-based immunocytochemistry and optogenetics
Source: Brain Struct Funct. 2020 Nov 9;226(1):105–20. doi: 10.1007/s00429-020-02167-7 (PMC7817561; doi:10.1007/s00429-020-02167-7)
Supplement: Supplementary file 1 — Supplementary file1 (PDF 150 KB) [file 429_2020_2167_MOESM1_ESM.pdf]

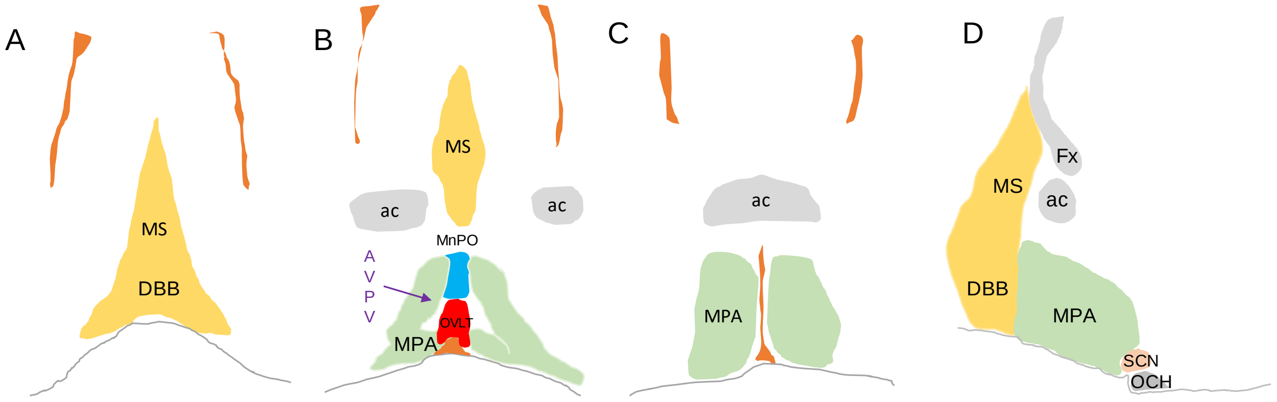

Supplementary Fig. 1. Schematic drawings illustrating the rostro-caudal segment of the mouse brain analyzed for networking of hypophysiotropic GnRH neurons and GLP-1 axons. **A-C**: coronal schemes, **D**: para-median sagittal scheme. ac: anterior commissure, AVPV: antero-ventral periventricular nucleus, DBB: diagonal band of Broca, Fx: fornix, MnPO: median preoptic nucleus, MPA: medial preoptic area, MS: medial septum, OCH: optic chiasm, OVLT: vascular organ of lamina terminalis, SCN: suprachiasmatic nucleus. Ventricular system is depicted in brown. A: 1 mm anterior to bregma. B: 0.5 mm anterior to bregma. C: 0.1 mm anterior to bregma. D: 0.2 mm lateral to midline.

Sample	Target	Reporter	Quencher	RQ	Cт	Cт Mean	Genotype
236954	cnr	VIC	TAMRA		23.03887	23.03887	
236954	cre	FAM	NFQ-MGB	2.075108	22.7028	22.7028	c/+
236955	cnr	VIC	TAMRA		23.58289	23.58289	
236955	cre	FAM	NFQ-MGB		Undetermined		+/+
236956	cnr	VIC	TAMRA		23.31219	23.31219	
236956	cre	FAM	NFQ-MGB	4.123183	21.98554	21.98554	c/c
236957	cnr	VIC	TAMRA		23.63722	23.63722	
236957	cre	FAM	NFQ-MGB		Undetermined		+/+
236958	cnr	VIC	TAMRA		23.49401	23.49401	
236958	cre	FAM	NFQ-MGB	1.992268	23.21672	23.21672	c/+
236959	cnr	VIC	TAMRA		24.39028	24.39028	
236959	cre	FAM	NFQ-MGB	4.245447	23.02147	23.02147	c/c
236960	cnr	VIC	TAMRA		23.77357	23.77357	
236960	cre	FAM	NFQ-MGB	3.738704	22.58815	22.58815	c/c
236961	cnr	VIC	TAMRA		24.15881	24.15881	
236961	cre	FAM	NFQ-MGB	3.714258	22.98285	22.98285	c/c
236962	cnr	VIC	TAMRA		24.00488	24.00488	
236962	cre	FAM	NFQ-MGB	3.633559	22.86061	22.86061	c/c
236963	cnr	VIC	TAMRA		23.85875	23.85875	
236963	cre	FAM	NFQ-MGB	3.825732	22.64013	22.64013	c/c
236964	cre	FAM	NFQ-MGB	3.842652	23.01165	23.01165	c/c
236964	cnr	VIC	TAMRA		24.23664	24.23664	
236965	cre	FAM	NFQ-MGB	3.428575	22.90805	22.90805	c/c
236965	cnr	VIC	TAMRA		23.96854	23.96854	
236966	cre	FAM	NFQ-MGB	4.069218	22.44661	22.44661	c/c
236966	cnr	VIC	TAMRA		23.75425	23.75425	
cre 1k	cnr	VIC	TAMRA		23.38457	23.38457	
cre 1k	cre	FAM	NFQ-MGB	1	24.10169	24.10169	
cre 2k	cnr	VIC	TAMRA		23.32614	23.32614	
cre 2k	cre	FAM	NFQ-MGB	1.821669	23.17799	23.17799	

Supplementary Table 1. Genotyping results of the gcg-cre mouse line. The quantitative analysis of the real-time PCR data demonstrates the relative quantity (RQ) of the cre transgene in homo- (c/c) and heterozygous (c/+) mice. RQ is the relative quantity of the *cre* vs the endogenous control copy number used as reference in the multiplex PCR system. The RQ of the calibrator samples (cre 1k and 2k) are shown in the bottom of the table (*blue*).
